# Effects of a synthetic feline facial pheromone analogue (F3, Feliway^®^) on stress and cardiovascular parameters during clinical examination in cats: a prospective crossover study

**DOI:** 10.1186/s12917-026-05511-x

**Published:** 2026-05-26

**Authors:** Felipe Gaia de Sousa, Natália Souza Ferreira, Camila Ferreira de Oliveira, Sol Aurum dos Reis, Giovanni Naves Canta, Fabiana Silva Fádel Queiroz, Roberta do Carmo Teixeira, Camila Stefanie Fonseca de Oliveira, Cláudio Roberto Scabelo Mattoso, Suzane Lilian Beier

**Affiliations:** 1 Department of Veterinary Clinic and Surgery, School of Veterinary Medicine, Federal University of Minas Gerais, 6627 Antônio Carlos Av, Pampulha, Belo Horizonte, Minas Gerais 31270-901 Brazil; 2 VETHEART – Cardiovascular Physiology and Veterinary Cardiology Research Group, Belo Horizonte, Brazil; 3 National Institute of Science and Technology in Nano-Biopharmaceutics (NANO- BIOFAR), Belo Horizonte, Brazil; 4 CargioVet – Specialized Service of Veterinary Cardiology, Belo Horizonte, Brazil; 5 Department of Preventive Veterinary Medicine, Veterinary School, Federal University of Minas Gerais – UFMG, Belo Horizonte, Brazil

**Keywords:** Echodopplercardiography, Feline pheromone, Hemodynamic changes, Stress, Undesirable behavior

## Abstract

**Background:**

Veterinary examinations in cats are essential for clinical assessment; however, stress often interferes with handling and data reliability. This study evaluated the cardiovascular and behavioral effects of a synthetic feline facial pheromone (F3) during cardiovascular examinations. A prospective repeated-measurements study was performed with 21 healthy mixed-breed cats over one year of age, non-aggressive and free of comorbidities. Each cat was assessed without pheromone exposure (T0) and after exposure (T1) using diffuser and spray formulations. Cats were transported in carriers and allowed a 20-minute acclimation period under controlled environmental conditions. Stress was assessed by two observers, followed by blood pressure measurement and cardiovascular evaluation. The second assessment occurred within 20 days, with the room prepared 48 h in advance with the pheromone analogue to minimize potential sequence or carry-over effects. Procedures were standardized and conducted by the same team. Statistical analyses compared time points and sexes and correlations between stress and cardiovascular variables with significance at *p* < 0.05.

**Results:**

All animals were clinically healthy, mean age of 5.73 ± 1.98 years, body weight of 5.41 ± 1.44 kg, and a majority being female. No significant differences were observed between temperature, heart rate (HR), respiratory rate, or arterial pressures (*p* > 0.05). The mean stress score decreased by 0.24 units at T1 (*p* < 0.05). Among electrocardiographic parameters, only maximum HR differed significantly between T0 and T1 (*p* < 0.05); all other parameters showed no significant differences. Significant differences were observed for ejection fraction (EF%), fractional shortening (FS%), A-wave velocity, and mitral annular plane systolic excursion (MAPSE) (*p* < 0.05). The number of animals with fused waves decreased and females showed a trend toward stress reduction, but without significance (*p* > 0.05). The S-wave amplitude, left ventricular internal diameter in systole (LVIDs), and EF% decreased only in males (*p* < 0.05). Both sexes showed differences in SF% and MAPSE (*p* < 0.05). No significant associations were found between stress and cardiovascular variables (*p* > 0.05).

**Conclusions:**

The synthetic analogue pheromone shows potential stress-related effects, may provide short-term cardiovascular benefits, no adverse effects, and may support cardiovascular assessments in healthy cats.

**Supplementary Information:**

The online version contains supplementary material available at 10.1186/s12917-026-05511-x.

## Background

Complementary diagnostic tests have become increasingly valuable tools for both diagnosis and clinical monitoring in veterinary medicine. However, their implementation can be challenging. Major obstacles include patient restraint, correct positioning, long examination times, stress in the animal or owner, and previous negative experiences [1–4]. Stress and fear during veterinary visits or examinations are major sources of discomfort for both cats and their owners [4, 5]. These factors may alter physiological parameters, potentially leading to under- or overestimation of clinical findings, such as BP [5]. Home-based veterinary assessments have been suggested as an alternative [6]. Thus, particularly in cats, clinical, laboratory, and imaging evaluations may often present variations that compromise the assessment of overall health status. The impact of stress on animal well-being underscores the need for strategies to ensure calmness and comfort during examinations [2, 5]. In addition to pre-appointment stress in cats, there is also a risk of stress in owners, who may experience some reluctance to bring their animals to veterinary consultations [2–4].

In addition to cat-friendly handling and restraint strategies based on CatFriendly practices [2, 5], and with the aim of using chemical/pharmacological restraint only as a last resort, substances that promote sensations of safety and comfort can be useful and cost-effective tools [7–12]. Analogues of feline facial pheromones, such as F3, derived from feline facial secretions [13], can help cats remain calm and secure during outings and visits to unfamiliar environments. These pheromones are associated with well-being and facilitate adaptation to people, other animals, and objects in the environment [14].

Studies on cardiovascular and behavioral effects of the synthetic feline facial pheromone analogue (F3) in cats are scarce and inconsistent. Several studies highlight the benefits of using the synthetic analogue of the feline facial pheromone for behavioral issues, such as undesirable scratching [13] as well as for controlling and/or reducing stress during veterinary assessments or transportation [15–18]. However, some studies found no effect on blood pressure measured at the tail [16] or limited behavioral effects in veterinary settings, often due to small sample sizes [19].

To date, this is the first study to evaluate F3 with detailed cardiovascular assessment, including electrocardiography and echodopplercardiography. We hypothesized that: (1) the use of the synthetic analogue of the feline facial pheromone (F3) improves the quality of cardiovascular examinations, particularly Doppler echocardiography; (2) the analogue significantly affects hemodynamic variables associated with fear and stress; and (3) the analogue provides comfort and reduces stress in cats following its application. The objective of the present study was to compare and evaluate the cardiovascular and behavioral effects of using the synthetic analogue of the feline facial pheromone (F3) formulated as an environmental diffuser and spray in healthy cats.

## Methods

### Ethical approval

This study, including all procedures involving the animals, was approved by the Ethics Committee on Animal Use (CEUA) of the Federal University of Minas Gerais (UFMG), Belo Horizonte, Brazil (protocol number 301/2022). For participation in the study, the owners and/or legal guardians agreed and signed the informed consent form. As described in the informed consent form, the owners and/or legal guardians had no financial cost with the study and could contact the research team if they wanted to acquire the results of the tests performed.

### Sample size

A priori sample size calculation was performed according to Miot [20] using the formula n = (Zα/2 × δ / E)², where n is the number of animals, Zα/2 is the critical value for 95% confidence (1.96), δ is the standard deviation of the variable of interest, and E is the maximum allowable error. Based on LVIDd (left ventricular end-diastolic dimension) reported by Fries et al. [21] as 15.3 ± 1.1 and considering a maximum error of 3% of the mean, the required sample size was calculated and rounded to 22 cats. A post hoc power analysis indicated a statistical power of approximately 60% to detect moderate effects for the primary variables, reflecting a moderate likelihood of type II error.

### Animals

Apparently healthy cats from veterinary clinics, owners and/or legal guardians, and non-governmental organizations were selected to participate in the present study between August and October 2023. A total of 22 cats of varying sexes and breeds were included, comprising 11 males and 11 females, all neutered, with variable body weights and age ranges. Inclusion criteria for participant selection were: age over 1 year, tolerance to handling, non-aggressive behavior, and absence of comorbidities such as cardiomyopathies (e.g., hypertrophic cardiomyopathy phenotype), cardiorenal syndrome, systemic and/or pulmonary arterial hypertension, chronic kidney disease, and zoonoses (e.g., sporotrichosis). Screening for retroviruses, feline immunodeficiency virus (FIV) and feline leukemia virus (FeLV), was performed using the Vet FAST FIV/FeLV Combo Test (Bioclin, Minas Gerais, Brazil); cats that tested positive but were otherwise asymptomatic were included in the study. Cats showing clinical abnormalities during examination, such as dehydration, diarrhea/vomiting, infectious diseases, or skin lesions, were also excluded. No anesthetic or sedative agents were used; all animals remained conscious throughout the procedures.

### Study design and methodology

**Fig. 1:**
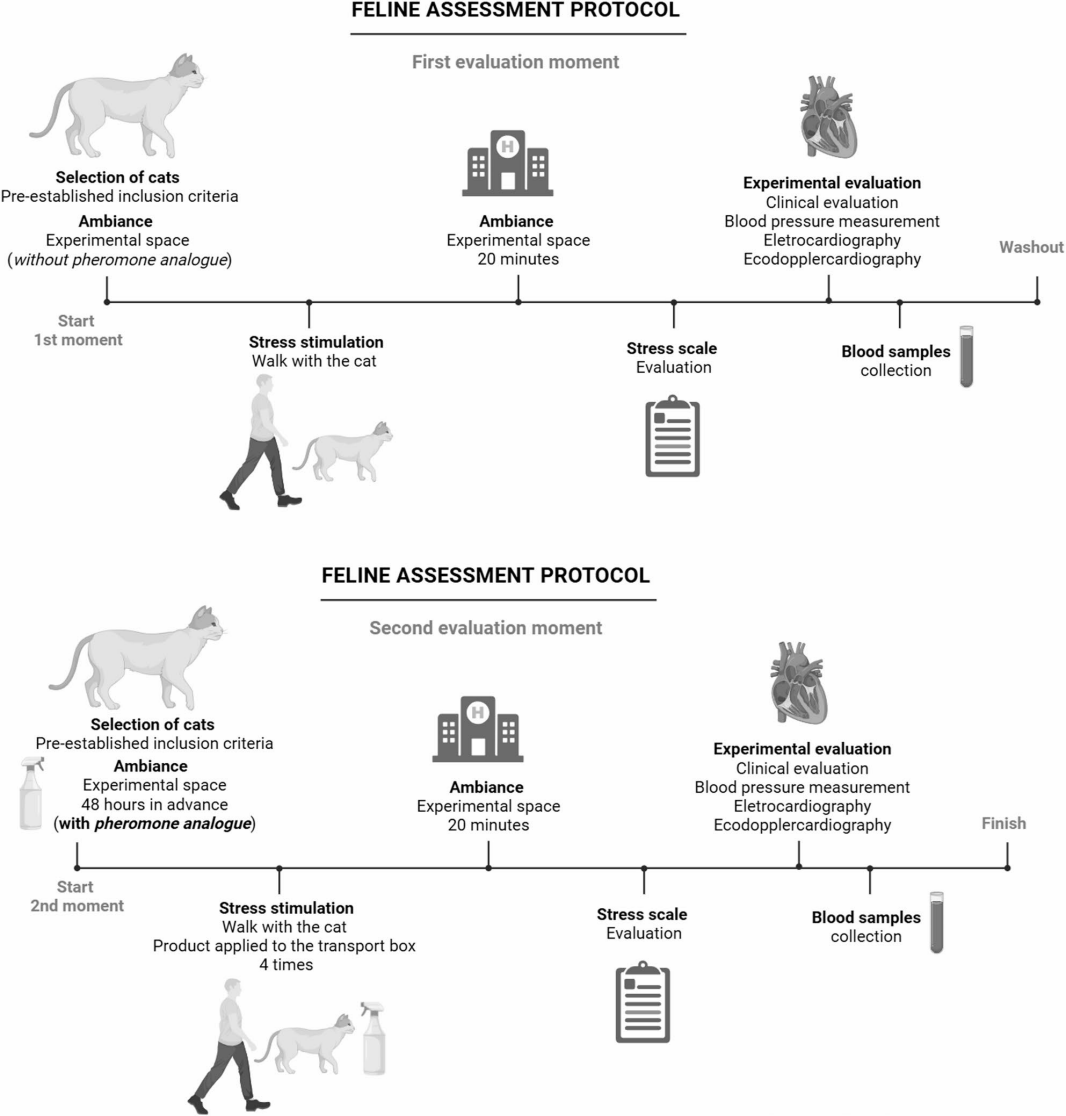
Experimental methodology for clinical and cardiovascular evaluation of cats before and after the use of the synthetic analogue of the facial feline pheromone fraction F3. Created by authors with BioRender.com

The study was designed as a prospective repeated-measures study with evaluations performed at two time points (T0 and T1). After selection according to pre-established criteria, the cats were brought to the facilities of the Veterinary Hospital of the Federal University of Minas Gerais (UFMG) for clinical evaluation by the responsible team at two distinct time points. The first (T0) and second (T1) evaluations were conducted by the same professionals to minimize methodological and procedural variability. The T0 assessment was based on clinical examination, imaging, and sample collection in an environment free of the synthetic analogue of the feline facial pheromone (F3) (Feliway, 60 ml, CEVA Saúde Animal Ltda). The second evaluation, T1, took place in an environment prepared with pheromone F3 48 h prior. Before each evaluation (T0 and T1), cats were allocated and allowed to acclimate for 20 min in the experimental room. Fig. 1 illustrates the experimental methodology for T0 and T1.

During the first evaluation (T0), conducted without the use of the synthetic feline facial pheromone analogue (F3) in diffuser or spray form, only cat-friendly handling practices were applied. Following completion of the animal registration forms, cats remaining in their transport carriers were subjected to a 250-meter transport within the carrier, guided by a single researcher. This procedure was intended to simulate transportation-related stress after arrival at veterinary facilities. The carriers were held in the researcher’s arms, and at no point was the animal carried by the carrier handle. The route was conducted within the Veterinary Hospital, exposing animals to typical noises such as barking dogs, vehicle sounds, and other environmental stimuli characteristic of a veterinary setting. After returning, cats were gently transferred to the experimental room for the 20-minute acclimation period.

Acclimation was performed by a single researcher, with the transport carrier containing the cat placed inside the procedure bay (80 × 60 × 100 cm³). Each animal had its own blankets and mats throughout the process. The carrier door/lid remained open during the entire acclimation period, and no human or other animal movement occurred to ensure calmness and minimize noise. Cats unwilling to exit the carrier voluntarily were carefully removed and placed on the evaluation table with positive reinforcement (e.g., treats or wet food). Following the acclimation period at T0, all 22 cats were assessed for stress by two researchers in the absence of their owners or legal guardians. Stress scoring was performed via observational assessment by two observers, with evaluators not communicating to avoid bias. The T0 evaluation served as the baseline, providing comparative data for the post-pheromone exposure assessment with each cat serving as its own control. Due to the nature of the intervention, blinding of observers was not feasible, as treatment conditions could be distinguished by detectable environmental odors.

At the conclusion of T0, BP measurements were performed by a single trained observer using dual methodology (vascular and oscillometric), with a cuff corresponding to 30–40% of the circumference of the left forelimb. Clinical evaluation was performed generally by assessing systemic clinical parameters (e.g., cardiorespiratory, locomotor, nervous, and genitourinary systems). All cats subsequently underwent Doppler echocardiography and electrocardiography performed by two trained observers, each assigned exclusively to one of the assessments, to obtain baseline cardiovascular data. One researcher performed echocardiography, and another performed electrocardiography. Blood samples were collected and submitted for laboratory analysis at the end of procedures. After T0, cats were returned to their owners.

For T1, the 21 cats were again brought to the Veterinary Hospital of UFMG within 20 days for the second phase to minimize potential sequence or carry-over effects. The experimental environment had been prepared 48 h prior with the synthetic feline facial pheromone analogue (F3) in diffuser form, which remained continuously powered throughout the study period. Upon arrival, a trained researcher removed each cat from its carrier, applied four sprays of the synthetic pheromone analogue (F3), waiting 15 min according to the manufacturer’s recommendations and returned the cat to the carrier.

**Fig. 2:**
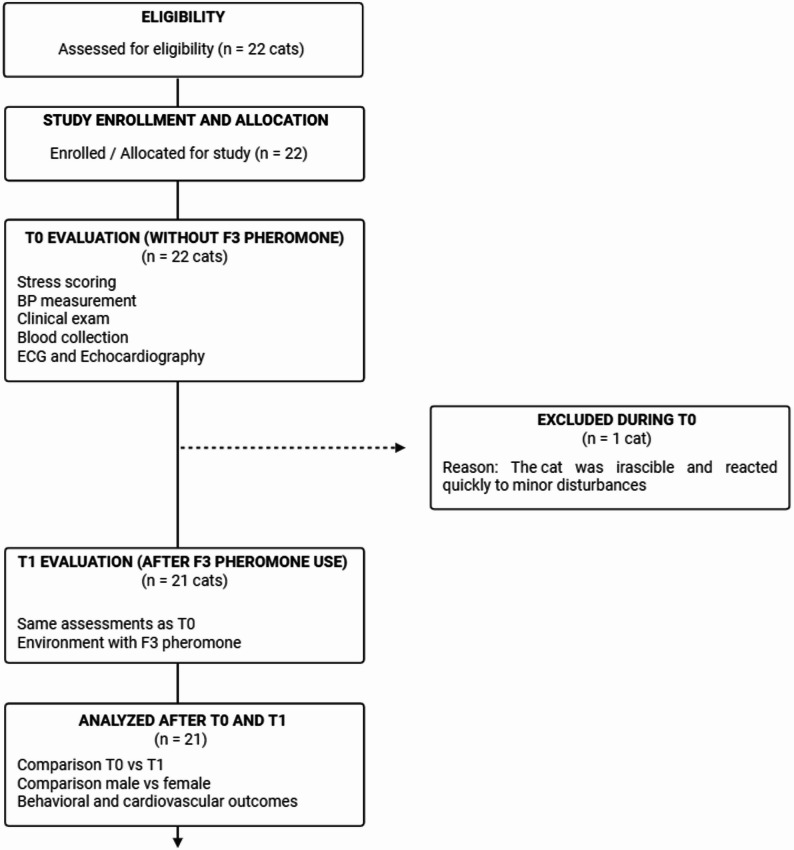
CONSORT-style flow diagram showing enrollment, exclusion, and completion of 22 cats in the study. One cat was excluded during T0 due to a clinical abnormality. The remaining 21 cats completed T1 and were included in the final analysis

Cats were then transported 250 m on foot within the carrier, similarly to T0, and subsequently placed in the experimental room for the acclimation period. All procedures at T1, including stress scoring, BP measurement, clinical, imaging, and laboratory assessments, were performed in the same manner as at T0. Upon completion of T1, cardiovascular and behavioral outcomes from the two evaluations were analyzed and compared. The flow of cats through the study, including enrollment, exclusion, and completion at baseline (T0) and post-intervention (T1), is shown in Fig. 2.

### Assessment of the stress score

Stress was assessed using the validated Cat Stress Score of Kessler & Turner, 1997 [22] at T0 and T1 by two trained observers. Dual observation was performed to minimize under- or overestimation of the obtained values, and observers did not communicate with each other to reduce potential bias. As previously described, blinding of observers was not feasible. The stress scale categorizes behavior into seven levels, ranging from fully relaxed to terrified. Cats were evaluated while inside their transport carrier with the door open, allowing voluntary exit, which ensured a standardized assessment context. The stress scale is currently the only validated instrument for assessing acute stress in cats, and no alternative validated tools were identified.

### Collection and evaluation of biological samples

Biological blood samples were collected after BP measurement, at a designated location using specific materials during both evaluations (T0 and T1). Blood collection was performed via radial vein venipuncture using a 23G scalp needle and a 5 mL syringe (Descarpack^®^, Luer Lock, Santa Catarina, Brazil). Obtained blood samples were transferred and stored in vacuum plastic tubes containing ethylenediaminetetraacetic acid (EDTA – purple top, 5 mL, BD Vacutainer^®^) for hematological analysis, and in tubes with clot activator (red top, 5 mL, BD Vacutainer^®^) for biochemical analysis, with subsequent submission to the laboratory for processing.

### Assessment of the clinical parameters

Clinical evaluation was conducted during both assessments, T0 and T1, with animals handled carefully according to cat-friendly handling principles. Due to the stainless-steel surface of the examination table, a rubber mat was placed to prevent direct contact between the animal and the table, along with the use of the cat’s own blankets to maintain olfactory familiarity. Clinical parameters were assessed via dual observation by two trained researchers performing systemic analysis, with final values obtained as the mean of both observers’ measurements. The variables analyzed included mucous membrane color, capillary refill time, heart (HR) and respiratory rates (RR) by auscultation, hydration status, body and muscle condition scores [23, 24], rectal temperature, lymph nodes, arterial pulse, and the corresponding physiological systems.

### Non-invasive blood pressure assessment

Non-invasive BP assessment was performed in the cats after the acclimation period, using the left forelimb with a cuff corresponding to 30–40% of the limb circumference. Ear protection was used during all measurements. The final BP value was calculated as the mean of seven readings obtained by a trained observer using both the Doppler vascular method (812B, Parks Medical, Beaverton, USA) and the oscillometric method (BPScan, InPulse Animal Health^®^, Florianópolis, Brazil), with the first and last readings discarded. Reference values were based on the guidelines of the International Society of Feline Medicine Consensus [25].

### Electrocardiography

During both T0 and T1 assessments, following the acclimation period, electrocardiographic examinations were performed using a 12-lead InCardio X device (InPulse Animal Health^®^, Florianópolis, Brazil). Leads I, II, III, aVL, aVR, aVF, and V1–V6 were used as previously described [26]. Electrocardiographic tracings were recorded for a minimum of 3 min using a computerized electrocardiography program for subsequent analysis.

### Echodopplercardiographic evaluation

Echocardiographic examinations were performed prior to and following the intervention (T0 and T1) to evaluate potential effects of the synthetic analogue of the feline facial pheromone (F3) on echocardiographic variables. During both assessments, cats were acclimated and placed in right and left lateral recumbency on a solid examination mat for echocardiography. Examinations were conducted without simultaneous electrocardiography; systolic and diastolic phases were determined visually based on valve motion and chamber dimension changes. The SonoSite Edge II device (Fujifilm, São Paulo, Brazil) with a sector transducer operating at 4.0–8.0 MHz was used for all examinations. Echocardiographic assessments at T0 and T1 were performed by a single trained observer. Left ventricular internal dimensions during diastole and systole (LVIDd and LVIDs), fractional shortening (FS%), and ejection fraction (EF%) were obtained using M-mode echocardiography from the right parasternal short-axis view, following the leading-edge convention. Left ventricular posterior wall and interventricular septum thickness (LVPWd, LVPWs, IVSd, IVSs) were measured in the same view using M-mode. Mitral E and A waves and the E/A ratio were measured using pulsed-wave Doppler at the mitral valve tips, while E′ and A′ waves and their ratio were assessed using tissue Doppler imaging at the lateral and septal mitral annulus. Isovolumetric relaxation time (IVRT), aortic (Ao) and left atrial (LA) diameters, and the left atrium-to-aorta ratio (LA/Ao) were measured using two-dimensional imaging in the right parasternal short-axis view at the level of the aortic valve, following the leading-edge convention. Pulmonary and aortic flow velocities and pressure gradients were measured using pulsed- and continuous-wave Doppler, and mitral annular plane systolic excursion (MAPSE) was assessed in M-mode at the lateral mitral annulus. Due to E/A wave fusion, some diastolic function parameters could not be reliably measured in all animals. Thus, cats with fused waves were excluded only from the corresponding analyses, which were performed using complete-case analysis. No imputation was applied because missingness reflected physiological/technical measurement limitations rather than random loss of data. As a sensitivity analysis, baseline characteristics were compared between cats with and without E/A wave fusion, and no marked differences were observed.

### Statistical analyses

Statistical analyses were performed using RStudio (version 4.1.0). The distribution of variables was assessed using the Shapiro–Wilk test, complemented by visual inspection through histograms and boxplots. For the entire group, comparisons between pre- and post-intervention measurements were performed using the paired t-test for normally distributed data (*p* > 0.05) and the Wilcoxon signed-rank test for non-normally distributed data (*p* < 0.05). Categorical variables in paired comparisons were analyzed using McNemar’s Chi-square test. When the objective was to evaluate differences between males and females, comparisons were made separately for pre- and post-intervention data. In the analyses involving differences between males and females, comparisons were conducted both at each moment (T0 and T1) and in relation to the change over moment (Δ = T1 – T0), in order to determine whether there was a difference in the increase or decrease after analogue synthetic pheromone exposure between sexes. For continuous variables, Welch’s t-test was applied to normally distributed data, and the Wilcoxon rank-sum test was used for non-normally distributed data. Categorical variables were compared using Pearson’s Chi-square test with continuity correction. Interobserver agreement for stress scoring was assessed using Cohen’s kappa coefficient with quadratic weighting. For multiple comparisons, the Bonferroni correction was applied, adjusting the significance level to 0.05/number of comparisons, in order to reduce the probability of false-positive results arising from multiple tests. Correlation analyses were conducted using Pearson’s correlation coefficient for normally distributed variables and Spearman’s rank correlation for non-normally distributed variables, in order to explore the relationship between stress and sedation and their potential effects on the outcomes. All results are presented in tables and figures. A supplementary table (Supplementary 1) was included to detail the number of animals analyzed for each variable. The significance level was set at 5% (*p* < 0.05).

## Results

### Demographic and clinical characteristics of the sample

Of the 22 cats initially enrolled, only 21 completed the clinical evaluations; one male was excluded because it did not permit completion of any assessments. Of these, 52% were female (*n* = 11/21) and 48% male (*n* = 10/21), with no significant differences between sexes (*p* > 0.05). The mean age was 5.73 ± 1.98 years, ranging from 3 to 9 years. All cats were of mixed breed. The mean body weight was 5.41 ± 1.44 kg, with a minimum of 3.10 kg and a maximum of 8.30 kg. Regarding retroviral status, all animals tested negative for FIV, and only 9.52% (*n* = 2/21) were positive for FeLV. No cats presented with cardiac murmurs during either clinical evaluation. In all assessments, mucous membranes were normocolored, pulses were normal in strength, and paw pads were intact, pink, adequately perfused, free of pain, and smooth/uniform in texture. All cats were clinically healthy based on laboratory results, including complete blood count and serum biochemistry, which included liver and kidney function tests. The majority of evaluated cats exhibited dental tartar and mild periodontal alterations (e.g., gingival erythema) (*n* = 16/21; 76.19%). Median body condition score and muscle condition score were 5 (range 1–9) and 3 (range 1–3), respectively. No animals presented clinical alterations that would preclude their participation in the study.

### Comparative analysis of all cats at T0 and T1

**Fig. 3:**
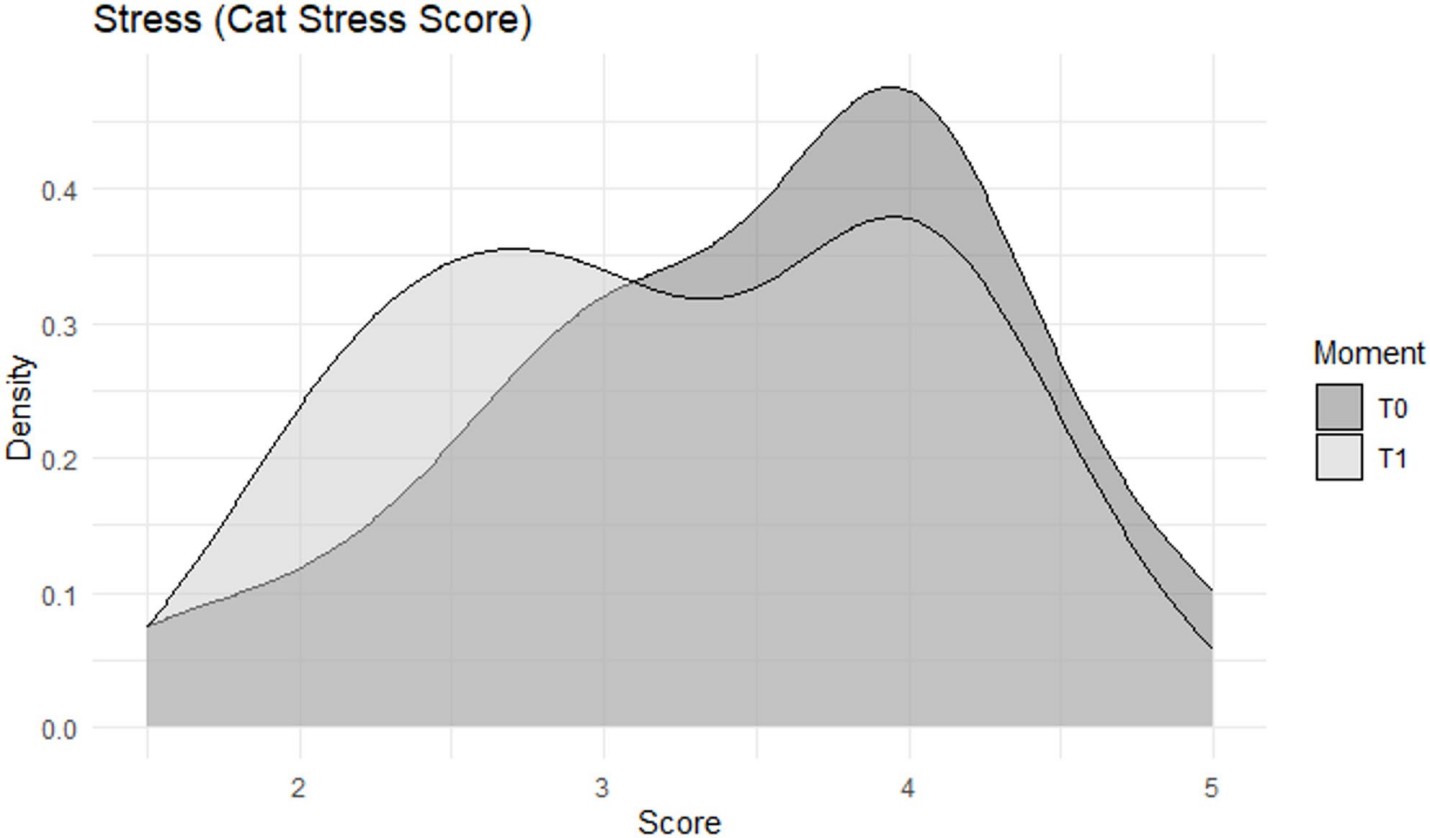
Comparison of stress scores in all cats evaluated at time points T0 (without the synthetic analogue of the feline facial pheromone – F3) and T1 (with the synthetic analogue of the feline facial pheromone – F3). Cats at T1 exhibited significantly lower stress scores compared to those at T0 (*p* < 0.05)

Regarding body temperature, HR (auscultation), and RR at T0 and T1, no significant differences were observed between the two time points (*p* > 0.05) (Table 1). No significant differences were detected in systolic arterial pressure (SAP) measured by the Doppler vascular method, or in SAP, mean arterial pressure (MAP), and diastolic arterial pressure (DAP) measured by the oscillometric method between the two assessments (*p* > 0.05) (Table 1). SAP values obtained by both methods were compared, but no significant differences were found (*p* > 0.05). Due to the exclusion of animals with incomplete or inconsistent laboratory data, the analysis was performed on 12 animals. No statistically significant differences were detected between T0 and T1 (Table 1). For the stress variable, both observers demonstrated agreement in the scores assigned, with no significant differences between them (*p* > 0.05) (Table 1). The mean stress score decreased by 0.24 units at T1 compared to T0 (*p* < 0.05; Fig. 3). Interobserver agreement was evaluated using Cohen’s kappa (quadratic weights). The control group showed κ = 0.671 (*p* = 0.002), whereas the treatment group showed κ = 0.884 (*p* < 0.001), indicating higher agreement in the treatment group.

**Table 1:** Distribution of feline data related to clinical, blood pressure and stress assessments in all moments described (T0 and T1)

Variable	Moment	Mean	Median	Q1	Q3	Min	Max	*p*
T (ºC)	T0	38.47^a^				37	39.80	0.5808
	T1	38.54^a^				37.10	39.80	
Body score (1–9)	T0		5^a^	4	6	4	7	0.1294
	T1		6^a^	4	7	3	7	
Muscle score (1–3)	T0		3^a^	3	3	2	3	0.3458
	T1		3^a^	3	3	3	3	
HR (bpm)	T0		196^a^	180	204	96	250	0.3976
	T1		184^a^	156	204	128	216	
RR (mpm)	T0	53.45^a^				32	80	0.5825
	T1	50.18^a^				26	72	
SAP Doppler (mmHg)	T0		140^a^	120	160	105	260	0.5626
	T1		150^a^	140	170	100	180	
SAP Oscillometric (mmHg)	T0	136.30^a^				91	205	0.5734
	T1	132.90^a^				96.50	184	
MAP Oscillometric (mmHg)	T0	120.30^a^				76	185	0.7893
	T1	118.60^a^				80.50	171	
DAP Oscillometric (mmHg)	T0	110.40^a^				68	175	0.8225
	T1	109^a^				70.50	162.50	
Hematocrit (%)	T0	44.06^a^				36.20	51.10	0.3962
	T1	42.52^a^				37.10	49.70	
Total leukocytes (×10³/µL)	T0		8500^a^	6125	11,050	4500	21,200	0.4798
	T1		8300^a^	6050	10,025	3300	11,800	
Segmented neutrophils (/µL)	T0		5656^a^	4558	7291	2790	10,000	0.4697
	T1		5104^a^	4352	7100	2343	9558	
Eosinophils (/µL)	T0		413^a^	157.5	595	0	1411	0.8501
	T1		423.50^a^	310.50	555	118	1248	
Lymphocytes (/µL)	T0	1897^a^				285	4484	0.5083
	T1	1699^a^				102	3663	
Monocytes (/µL)	T0		106^a^	54	241.50	0	354	0.9687
	T1		114.50^a^	0	283	0	560	
Platelets (×10³/µL)	T0		206^a^	139.20	289.20	48	672	0.1165
	T1		122^a^	90.25	213	73	452	
Glucose (mg/dl)	T0	86.81^a^				47.43	158.26	0.6728
	T1	94.37^a^				51.36	117.24	
Stress scale (1–7)	T0	3.47^a^				1500	5000	0.0291*
	T1	3.23^b^				2000	4500	

*Abbreviations: T0 Control moment, *T1* Pheromone analogue moment, *Q1* First quartile, *Q3* Third quartile, *HR* Heart rate, *RR* Respiratory rate, *SAP* Systolic arterial blood pressure, *MAP* Mean arterial blood pressure, *DAP* Diastolic arterial blood pressure

^a, b^Different superscript letters in the column indicate statistical differences for comparison of mean (Paired t test) or median (Wilcoxon signed-rank test) between moments

*Significant at the 5% level

Regarding electrocardiographic variables, no significant differences were observed between T0 and T1 for P-wave amplitude and duration, PR interval duration, Q-, R-, S-, and T-wave amplitudes, R-wave duration, or ST-segment deviation and duration (*p* > 0.05) (Table 2). Considering the minimum and mean HR obtained from the electrocardiogram, no significant differences were detected (*p* > 0.05) (Table 2). However, a significant difference was observed for maximum HR between the two time points (*p* < 0.05) (Table 2). Maximum HR by electrocardiogram decreased significantly at T1 compared to T0 by 23.6 bpm, corresponding to a 9.02% reduction (T0: 261.9 bpm vs. T1: 238.3 bpm; *p* < 0.05).

**Table 2:** Distribution of feline electrocardiographic measurements in each moment evaluated (T0 – without pheromone analogue; T1 – with pheromone analogue). The values are expressed with mean or median

Variable	Moment	Mean	Median	Q1	Q3	Min	Max	*p*
P wave amplitude (mV)	T0	0.07^a^				0.02	0.11	0.4440
	T1	0.07^a^				0.04	0.13	
P wave duration (ms)	T0	33.14^a^				22	40	0.9564
	T1	33.24^a^				22	44	
PR interval (ms)	T0	68.24^a^				44	96	0.7619
	T1	68.81^a^				50	89	
Q wave amplitude (mV)	T0		−0.01^a^	−0.02	0	−0.14	0.01	0.2554
	T1		−0.02^a^	−0.05	−0.01	−0.11	0.04	
R wave amplitude (mV)	T0	0.22^a^				0.01	0.58	0.0852
	T1	0.16^a^				−0.11	0.42	
R wave duration (ms)	T0	44.05^a^				28	57	0.1670
	T1	47.48^a^				30	74	
S wave amplitude (mV)	T0		0.00^a^	−0.01	0.03	−0.05	0.07	0.1351
	T1		0.01^a^	−0.03	0.03	−0.25	0.06	
ST segment deviation (mV)	T0	0.01^a^				−0.02	0.06	0.3771
	T1	0.01^a^				−0.06	0.04	
ST interval duration (ms)	T0	60.67^a^				19	102	0.2581
	T1	66.67^a^				44	98	
T wave amplitude (mV)	T0	0.01^a^				−0.06	0.11	0.2305
	T1	0.04^a^				−0.10	0.22	
T wave duration (ms)	T0		34^a^	28	40	24	70	0.1352
	T1		39^a^	33	61	12	78	
Minimum HR (bpm)	T0	155.80^a^				84	218	0.3019
	T1	148^a^				102	192	
Mean HR (bpm)	T0		190^a^	166	204	116	258	0.7367
	T1		178^a^	164	200	156	258	
Maximum HR (bpm)	T0	261.90^a^				172	338	0.0376*
	T1	238.30^b^				170	320	

*Abbreviations: T0* Control moment, *T1* Pheromone analogue moment, *Q1 and Q3* First and third quartile, *HR* Heart rate

^a, b^Different superscript letters in the column indicate statistical differences for comparison of mean (Paired t test) or median (Wilcoxon signed-rank test) between moments

*Significant at the 5% level

Regarding echocardiographic variables, no significant differences were observed for IVSd, IVSs, LVFWd, LVFWs, LVIDd, LVIDs, Ao and LA diameters, LA/Ao, pulmonary flow and gradient, aortic flow and gradient, mitral E/EA wave, E-wave deceleration, lateral E′ and A′ waves and their ratio, and IVRT (*p* > 0.05) (Table 3). Regarding E/A wave fusion, 10 cats (47.6%) exhibited fused waves at T0, compared to 6 cats (28.6%) at T1; however, the difference in proportions was not statistically significant (*p* = 0.3428) (Table 3). The EF% and FS% decreased significantly at T1 by 6% and 7%, respectively (*p* < 0.05). A significant difference between time points was noted for the A-wave (*p* < 0.05) (Table 3). Additionally, MAPSE showed a significant reduction at T1, with a median value 0.18 cm lower than T0 (*p* < 0.05) (Table 3). For A-wave, A′-wave, and E-wave deceleration variables, analysis was performed on only 11 cats due to wave fusion (Table 3). Because E/A wave fusion reduced the number of cats with measurable values for some diastolic variables, a sensitivity analysis was performed by comparing baseline characteristics between cats with and without fused waves. No marked baseline differences were observed between these groups. Nevertheless, because only 11 cats had measurable values for variables such as A-wave, E/A ratio, E-wave deceleration time, A′ wave, and E′/A′ ratio, results for these parameters should be interpreted with caution.

**Table 3:** Distribution of feline echodopplercardiographic assessment in each moment evaluated (T0 or T1 without or with pheromone analogue). The values are expressed with mean or median

Variable	Moment	Mean	Median	Q1	Q3	Min	Max	*p*
IVSd (cm)	T0		0.38^a^	0.35	0.46	0.30	0.58	0.6725
	T1		0.38^a^	0.35	0.46	0.30	0.64	
IVSs (cm)	T0	0.74^a^				0.54	0.88	0.6882
	T1	0.73^a^				0.54	0.98	
LVWTd (cm)	T0	0.47^a^				0.30	0.50	0.6007
	T1	0.46^a^				0.30	0.54	
LVWTs (cm)	T0	0.73^a^				0.50	0.88	0.3069
	T1	0.71^a^				0.54	0.96	
LVIDd (cm)	T0	1.42^a^				1.04	1.69	0.0792
	T1	1.35^a^				1	1.65	
LVIDs (cm)	T0		0.62^a^	0.54	0.70	0.46	0.81	0.1311
	T1		0.65^a^	0.54	0.81	0.46	0.86	
EF (%)	T0		88^a^	86	92	59	95	0.0189*
	T1		82^b^	78	86	73	94	
SF (%)	T0	55.80^a^				40	66	0.0019*
	T1	48.80^b^				37	64	
Ao diameter (cm)	T0	0.84^a^				0.66	1.14	0.4954
	T1	0.86^a^				0.73	1.14	
LA diameter (cm)	T0		1.09^a^	1.03	1.21	0.89	1.44	0.8755
	T1		1.12^a^	1.04	1.16	0.96	1.48	
LA/Ao ratio	T0	1.35^a^				1.15	1.52	0.5371
	T1	1.33^a^				1.12	1.68	
PA Vel max (m/s)	T0	0.84^a^				0.66	0.98	0.4166
	T1	0.86^a^				0.69	1.05	
PA PG (mmHg)	T0	2.84^a^				1.75	3.90	0.4227
	T1	3.02^a^				1.94	4.45	
Ao Vel max (m/s)	T0	0.91^a^				0.74	1.18	0.7333
	T1	0.90^a^				0.68	1.10	
Ao PG (mmHg)	T0	3.41^a^				2.19	5.65	0.6927
	T1	3.29^a^				1.87	4.84	
E-wave or EA-wave (m/s)	T0	0.80^a^				0.58	0.98	0.1109
	T1	0.75^a^				0.55	0.93	
E-wave deceleration (ms)	T0		63^a^	60	76	59	100	0.2500
	T1		72^a^	68.75	82.50	55	100	
A-wave (m/s)	T0	0.62^a^				0.49	0.78	0.0495*
	T1	0.55^b^				0.39	0.70	
E-wave/A-wave ratio	T0	1.23^a^				0.77	1.55	0.4324
	T1	1.35^a^				0.86	1.74	
E’ septal (ms) or EA’ ratio	T0		0.08^a^	0.07	0.09	0.06	0.10	0.9543
	T1		0.08^a^	0.07	0.08	0.05	0.70	
A’ septal wave (m/s)	T0		0.07^a^	0.06	0.07	0.05	0.09	1
	T1		0.06^a^	0.06	0.07	0.05	0.50	
E’/A’ septal wave	T0	1.12^a^				0.75	1.67	0.5667
	T1	1.19^a^				0.71	1.43	
IVRT (ms)	T0	44.62^a^				37	55	0.0573
	T1	47.67^a^				40	60	
MAPSE (cm)	T0		0.69^a^	0.65	0.71	0.49	0.77	0.0003*
	T1		0.51^b^	0.45	0.60	0.38	0.63	

*Abbreviations: T0* Control moment, *T1* Pheromone analogue moment, *Q1 and Q3* First and third quartile, *IVSd/IVSs* Interventricular septum in diastole/systole, *LVIDd/LVIDs* Left ventricular intern diameter in diastole/systole, *LVWTd/LVWTs* Left ventricular wall thickness in diastole/systole, *EF% and FS%* Ejection and fractional shortening, *HR* Heart rate, *Ao* Aorta artery, *LA* Left atrium, *LA/Ao* Left atrium-aorta ratio, *PA* Pulmonary artery, *PG* Pressure gradient, *Vel max* Velocity, *Ao* Aorta artery, *IVRT* Isovolumetric relaxation time, *MAPSE* Annular mitral plane systolic excursion

^a, b^Different superscript letters in the column indicate statistical differences for comparison of mean or median between groups

*Significant at the 5% level

### Comparative stratification by sex of cats at T0 and T1

The same variables were analyzed stratified by sex, excluding from analysis those variables with incomplete data: A-wave, E/A ratio, A-wave deceleration, E′ wave, A′ wave, and E′/A′ ratio. No significant differences in age were observed between males and females (4.9 years vs. 6.5 years; *p* > 0.05). Body weight also did not differ significantly between sexes (5.81 kg vs. 5.05 kg; *p* > 0.05). During the experimental phase, nine males (*n* = 9/10; 90%) and seven females (*n* = 7/11; 63.64%) presented with dental tartar and periodontal disease, with no significant differences between sexes (*p* > 0.05). Retroviral status did not differ significantly between males and females for either FIV or FeLV.

For HR, RR, temperature, body condition score, muscle condition score, SAP measured by Doppler vascular method, and SAP, MAP, and DAP measured by oscillometric method, no significant differences were observed between sexes (*p* > 0.05). Regarding stress, females showed a trend toward reduced stress following pheromone exposure (*p* = 0.071), which did not reach statistical significance (*p* > 0.05). However, when comparing sexes, no significant differences were observed between males and females (*p* > 0.05).

For electrocardiographic variables, no significant differences were observed between males and females for P-wave amplitude and duration, PR interval duration, Q- and R-wave amplitudes, R-wave duration, ST-segment duration, T-wave amplitude and duration, and minimum, mean, and maximum HR (*p* > 0.05). However, a statistically significant difference was observed for S-wave amplitude between females and males (*p* = 0.022). Accordingly, the significant reduction in S-wave amplitude after the use of the synthetic feline facial pheromone analogue was observed exclusively in the male group, with the difference being significant (*p* = 0.032). Similar results were observed for most Doppler echocardiographic variables at T0 and T1. No significant differences between males and females were found for IVSd, IVSs, LVFWd, LVFWs, LVIDd, Ao and LA diameters, LA/Ao ratio, pulmonary and aortic flow and gradients, mitral E/A flow ratio, lateral E′ wave, and IVRT (*p* > 0.05).

For LVIDs, males showed a significant increase (*p* = 0.04206), although no difference was observed when compared with females (*p* > 0.05). The EF% decreased significantly only in males (*p* = 0.00005), with no difference between sexes (*p* > 0.05). The FS% decreased significantly in males after exposure to the synthetic feline facial pheromone analogue, with differences observed between males and females (*p* = 0.00319). Regarding MAPSE, pheromone exposure resulted in a significant reduction in systolic function in both males (*p* = 0.00003) and females (*p* = 0.02183), without differences between sexes (*p* > 0.05).

### Correlation analysis of stress with associated variables

Based on these findings, a correlation analysis was performed considering stress as the variable of interest and those variables that showed significant differences (*p* < 0.05) as associated: maximum HR, EF%, FS%, A-wave, and MAPSE. Correlation analyses did not reveal any significant associations between the variable of interest and the associated variables (*p* > 0.05) (Table 4).

**Table 4:** Pearson correlation coefficients comparing the association between the variable ‘stress score’ and other variables

Correlation with variable “stress score”		
Variables	Pearson’s rho	*p*
Maximum HR (electrocardiogram) (bpm)	0.24	0.2904
EF (%)	0.34	0.1354
FS (%)	0.30	0.1794
A-wave (m/s)	0.33	0.4274
MAPSE (cm)	−0.08	0.7192

*Abbreviations: HR* Heart rate, *EF%* Ejection fraction, *FS%* Fractional shortening, *MAPSE* Systolic excursion of the mitral annulus plane

*Significant at the 5% level

## Discussion

The results of this study suggest that the synthetic feline facial pheromone analogue – F3, in both diffuser and spray formulations, was associated with modest reductions in behavioral stress during cardiovascular examinations in apparently healthy cats aged 1–9 years and weighing 3–9 kg. No adverse effects were observed in this sample; however, the small cohort and lack of placebo control limit conclusions regarding cardiovascular safety and efficacy. These preliminary observations should be interpreted cautiously and require confirmation in future studies before clinical recommendations can be made. The selected sample consisted of a relatively homogeneous population of adult cats, with body weights ranging from ideal to overweight (body condition score 4–7) and adequate muscle mass. The male-to-female ratio was 10:11, with no significant differences between sexes. Regarding breed, all included cats were of mixed breed. The high prevalence of mixed-breed cats may be associated with factors such as adoption incentives, voluntary rescue efforts, and responsible ownership practices, particularly for vulnerable animals supported by public initiatives [27]. Regarding retroviruses, the majority of animals were FIV-negative, with a few cases of FeLV positivity but were asymptomatic, and showing no differences between males and females and indicating a low prevalence of these diseases in the evaluated sample. The potential influence of FeLV-positive status on the study outcomes was considered minimal due to the small number of affected cats. All included animals met the inclusion criteria, particularly regarding clinical and laboratory health status (complete blood count and serum biochemistry, including liver and kidney function assessment). Notably, 76.2% of the cats exhibited dental tartar and mild periodontal changes (e.g., gingival erythema), with no significant sex differences, which may be associated with factors such as diets and inadequate oral hygiene [28–30]. These conditions predispose to bacterial plaque accumulation and gingival inflammation, and in some cases may ultimately lead to tooth extraction [31]. Although these cats were considered clinically healthy, the presence of asymptomatic FeLV positivity and minor oral alterations should be taken into account when interpreting the findings, although their overall impact is likely minimal. It is important to note that all cats included in this study were mixed-breed domestic cats from a single geographic region. Therefore, the observed effects of the synthetic feline facial pheromone analogue F3 may not fully reflect responses in specific pure breeds. Only one cat was excluded due to being completely unmanageable. Although this study did not include cats with cardiovascular disease, stress-reducing interventions such as F3 exposure may hold potential for improving examination tolerance in clinical populations. Future studies should directly evaluate these effects in cats with cardiovascular conditions.

Another limitation of this study is that some diastolic echocardiographic variables were available only in a reduced subset of cats because E/A wave fusion prevented reliable measurement. Although baseline comparisons between cats with and without fused waves did not reveal marked differences, the smaller sample size for these variables may have reduced statistical precision and increased the risk of type II error. Therefore, findings related to these parameters should be interpreted cautiously and considered exploratory until confirmed in larger studies.

Regarding the effect of the synthetic feline facial pheromone analogue – F3 on animal stress, the mean stress score decreased by 0.24 units from T0 to T1 (*p* < 0.05; Table 1; Fig. 3). Although the decrease in mean stress score was modest, even small reductions may have clinical relevance in feline patients. Stress in this study was defined exclusively based on behavioral scoring, and cardiovascular variables were not used as primary indicators of stress. Cats are highly sensitive to stress during veterinary procedures, and minor improvements in stress scores can facilitate handling, reduce the risk of stress-induced physiological alterations, and improve the reliability of clinical assessments. Therefore, the observed reduction, while statistically small, may reflect a possible improvement in animal welfare during cardiovascular evaluations. Behavioral scoring alone may not fully capture the physiological stress response. No physiological stress biomarkers, such as serum cortisol, heart rate variability, or blood lactate, were measured in this study, and the reliance on behavioral scoring limits the biological interpretation of stress responses. It is important to note that, although a 20-day washout period was implemented between assessments, T1 evaluations occurred after cats had prior exposure to the same environment and procedures during T0. Therefore, some reduction in stress at T1 may reflect partial habituation or acclimation rather than the effects of the synthetic feline facial pheromone analogue (F3) alone. Environmental factors such as transportation, handling, and repeated exposure to the same clinical environment may also have influenced stress and cardiovascular parameters, making it difficult to attribute the observed changes solely to pheromone F3 exposure. This sequential, before–after design limits the ability to attribute observed effects solely to pheromone exposure. Additionally, as observers were not blinded to treatment conditions, the potential influence of observer perception on stress scoring cannot be completely excluded.

When animals were stratified by sex, a statistical trend was observed in females (*p* = 0.0707); however, no significant difference was found when compared with males (*p* > 0.05). Similar effects of stress reduction through the use of the synthetic feline facial pheromone – F3 have been reported by Pereira et al. [15], who described that cats exposed to the product exhibited lower stress scores and were more easily handled, suggesting its use in veterinary assessments, while also recommending evaluation of sex-specific effects. Other studies have reported various additional benefits, including control of scratching behavior, assistance during transport, reduction of urine marking, mitigation of inter-cat aggression, and improved coexistence with other species [13, 17, 32–34]. However, a recent study evaluated the influence of cats under the effects of pheromone, catnip, or 0.9% saline solution, with a 48-hour interval between tests and 10 min of exposure to each product [19]. The authors observed that the tested products had no effect on clinical or behavioral variables; however, the sample size was small (*n* = 8), the animals waited 10 min in the reception area prior to experimentation, and the exposure time to the products may have been too short.

From a clinical perspective, although HR assessed by cardiac auscultation appeared relatively high at both time points, this finding is commonly reported in cats undergoing clinical examination. Transportation, handling, physical restraint, and exposure to an unfamiliar environment may induce sympathetic activation and transient stress-related tachycardia in this species [6, 9]. In the present study, the absence of significant differences between T0 and T1 suggests that the pheromone diffuser did not substantially influence HR measured by auscultation under the conditions of the examination. In this study, auscultatory HR was not intended as a direct proxy of stress intensity, but as a clinical physiological parameter.

From an electrocardiographic perspective, considering the entire group of selected cats, no ECG variables showed significant differences between T0 and T1 (Table 2), except for maximum HR. Electrocardiogram-derived maximum HR decreased by approximately 9% (23 bpm) from T0 to T1 (*p* < 0.05; Table 2). However, correlation analysis did not reveal a significant association between this reduction and pheromone exposure (*p* > 0.05; Table 4). In healthy cats without echocardiographic abnormalities, electrocardiographic parameters, including maximum HR, may be influenced by methodological and recording variability, may be influenced by methodological and recording variability inherent to electrocardiographic acquisition in conscious cats rather than true physiological effects.

Regarding echocardiographic variables, most did not show significant differences between the two time points (Table 3). In the overall group, EF% and FS% decreased by approximately 6% and 7% at T1, respectively (*p* < 0.05; Table 3). When analyzed by sex, EF% decreased significantly in males but not females (*p* < 0.05). Decreases in EF% and FS% in healthy cats without evident cardiovascular abnormalities may be associated with stress. High sympathetic activation, particularly in alert or flight situations, can increase cardiac contractility, resulting in temporary elevations of EF% and FS%, especially during acute stress. Conversely, when animals are relaxed, with more pronounced parasympathetic activity, better cardiac relaxation occurs, leading to physiological reductions in EF% and FS%. Therefore, under conditions of acute stress, patients are subject to temporary, physiological hemodynamic changes due to increased sympathetic autonomic tone, which do not reflect disease or deleterious conditions. A study demonstrated that increases in HR can induce temporary elevations in FS%, which are directly proportional when a positive correlation is present [35]. Reduction in FS% following the use of sedative drugs has been reported with lower values observed post-sedation [36, 37].

Some studies have not demonstrated changes in EF% following the administration of gabapentin [10] or trazodone [36], although cardiovascular effects have been described with certain anesthetic combinations [38]. Studies evaluating gabapentin and trazodone in cats report reduced stress and improved compliance during clinical procedures, which may support the interpretation that reductions in stress can influence cardiovascular parameters; however, their effects appear to be variable [7, 8, 10]. While echocardiographic changes are generally minimal, some effects such as reduced arterial blood pressure or improved diastolic assessment may occur depending on the parameter evaluated [3, 10]. However, these drugs act through central nervous system modulation, differing substantially from the mechanism of action of synthetic pheromones such as F3. Therefore, comparisons between these approaches should be interpreted as indirect. Although stress scores decreased following pheromone F3 exposure, no direct physiological measures of autonomic activity were collected, and no significant associations with EF% or FS% were observed (*p* > 0.05; Table 4). Thus, interpretations regarding parasympathetic influence remain uncertain and should be approached cautiously.

Specifically in males, LVIDs showed a significant increase (*p* < 0.05), although no differences were observed when compared with females (*p* > 0.05). Under conditions of stress reduction and/or tachycardia control, animals may exhibit increases in LVIDs due to cardiac relaxation, allowing for improved stroke volume per cardiac cycle. Since the 1980s, Jacobs & Knight [39] already demonstrated a negative correlation between HR and LVIDs, showing reductions as cats exhibited tachycardia. A recent study highlighted the influence of tachycardia on LVIDs and LVIDd in cats, showing that increasing heart rate is associated with an increase in LVIDs and a concomitant reduction in end-diastolic diameter, indicating a positive correlation [35]. Similar effects were observed following the administration of dexmedetomidine and buprenorphine in cats, sedative drugs, where post-sedation measurements showed reductions in LVIDs [37]. However, other study comparing placebo versus trazodone, a drug with potential sedative effects, did not report changes in LVIDs even with dose variations [36]. Observed changes in LVIDs could reflect normal physiological variability or reduced stress, and cannot be interpreted as definitive evidence of direct cardiac effects of pheromone F3.

A condition frequently observed in cats experiencing temporary or acute stress is the fusion of the E and A waves, representing the rapid and slow filling phases of the left ventricle during the cardiac cycle. Wave fusion is derived from the stress process in cats during Doppler echocardiographic evaluations, often associated with tachycardia (> 170 bpm) and partial or complete alterations in wave morphology [10]. Wave fusion is considered an obstacle to precise diastolic assessment due to the inability to separate the waves for accurate measurement. It generally occurs under high HR conditions and can sometimes be managed, for example, with gabapentin [10]. Tachycardias above 180 bpm are associated with partial or complete fusion of LV filling waves [10]. In the present study, 47.6% of animals presented fused waves at T0, whereas only 28.6% did at T1, showing a 19% reduction; however, this difference was not statistically significant (*p* > 0.05). Although not statistically significant, this reduction in E/A wave fusion may suggest a potential improvement in diastolic filling, reflecting better relaxation of the left ventricle under conditions of lower stress. Consequently, some analyses were conducted only on cats without wave fusion during Doppler echocardiography. Regarding A-wave velocity, a significant difference was observed between T0 and T1 (*p* < 0.05; Table 3), indicating lower left ventricular filling pressure at T1 compared to T0, although no significant association was found (*p* > 0.05; Table 4). However, the interpretation of these diastolic variables (A-wave, E/A ratio, E-wave deceleration time, A′ wave, and E′/A′ ratio) requires caution. Due to E/A wave fusion, only a subset of cats (*n* = 11) had measurable values for parameters such as A-wave, E/A ratio, E-wave deceleration time, A′ wave, and E′/A′ ratio, and were therefore included in the analysis. Consequently, the observed findings, including the significant difference in A-wave, are based on a limited number of animals and may not adequately reflect consistent physiological changes across the entire study population. In this context, these results should be considered exploratory, definitive conclusions regarding diastolic function cannot be established, and should be confirmed in studies with larger sample sizes.

Concerning systolic function, MAPSE was 0.18 cm lower in cats at T1 (*p* < 0.05), with significant effects observed for both males and females (*p* < 0.05), but no differences between sexes (*p* > 0.05). MAPSE is an important marker of systolic function, reflecting mitral annular displacement [40]. Therefore, patients under temporary stress may exhibit higher MAPSE values which could be influenced by physiological and hemodynamic changes associated with arousal states, in the absence of disease or functional/structural cardiac abnormalities. Once temporary stress is controlled, these changes may be associated with different autonomic states. While previous research has shown that HR can influence MAPSE values [41], no significant HR differences were observed between T0 and T1 in this study. Therefore, any changes in MAPSE cannot be directly attributed to F3 exposure.

Thus, in the present study, animals assessed at T1, when the synthetic feline facial pheromone F3 was used, presented lower values compared to T0 (Table 4), although no significant association with stress was found (*p* > 0.05; Table 4). Based on these observations, exposure to the feline facial pheromone analogue was associated a modest behavioral effect under clinical examination conditions and minor changes in cardiovascular parameters in the absence of observed adverse effects. These preliminary findings suggest potential utility for supporting cardiovascular assessments, but should be interpreted cautiously pending further validation in larger, controlled studies.

It is important to note that the present study followed a sequential before–after repeated measures design rather than a randomized crossover trial. Cats were first evaluated without pheromone exposure (T0) and subsequently with pheromone exposure (T1), without a placebo condition. A 20-day washout period was implemented between the two assessments to minimize potential sequence or carry-over effects, although minor habituation or learning effects cannot be completely excluded. Therefore, potential confounders such as habituation to the environment, learning effects from prior examinations, decreased stress due to familiarity with procedures, and temporal bias may have contributed to the observed changes. While the study provides preliminary evidence of short-term cardiovascular benefits and stress reduction associated with F3 exposure, the absence of randomization and a placebo control limits the ability to attribute these effects solely to the pheromone. Future studies employing randomized, placebo-controlled designs are warranted to confirm these findings.

The present study has important methodological limitations. One limitation is the lack of observer blinding, which may have introduced observer bias, particularly in subjective assessments. Therefore, several outcome measures were operator-dependent (e.g., stress scoring and echocardiographic acquisition), which may have introduced measurement variability despite the use of trained evaluators and standardized procedures. Additionally, the non-randomized design and absence of a placebo control represent important methodological limitations. Although a 20-day washout period was implemented, behavioral conditioning could not be excluded, as cats may have partially habituated to the clinical environment or developed associations with prior procedures, potentially influencing physiological measurements during the second evaluation and attenuating or masking the effects of F3. The sample size may have influenced the observed results, highlighting the need for further studies with larger cohorts to confirm these effects more robustly. A post hoc power analysis indicated a statistical power of approximately 60% to detect moderate effects, suggesting a potential risk of type II error. Therefore, non-significant findings should be interpreted with caution and do not necessarily indicate equivalence between groups. A limitation of this study is the reduced sample size in some analyses, which may have decreased statistical power. Therefore, the absence of statistically significant differences should be interpreted with caution and does not necessarily indicate equivalence between groups. Additionally, despite the application of Bonferroni correction for multiple comparisons, significant findings should be interpreted carefully, as the combination of multiple comparisons and limited sample size may still influence the results. These findings should be considered exploratory and warrant confirmation in studies with larger sample sizes. One limitation was the absence of a comparison between the different formulations of the synthetic F3 pheromone (diffuser vs. spray), preventing isolation of their individual effects. Additionally, dose, environmental concentration, and exposure consistency were not quantified, which may have influenced the reproducibility of the findings.

Additional limitations should be considered. All cats were domestic shorthairs, limiting extrapolation of results to other breeds, and no comparisons were made regarding age-related effects. Subgroup analyses by sex further reduced statistical power, and therefore findings from these comparisons should be interpreted cautiously and considered exploratory. Although the overall clinical impact of asymptomatic FeLV positivity and mild periodontal alterations (e.g., gingival erythema) is likely minimal, their inclusion represents a potential limitation, and the small number of affected cats prevented separate analyses excluding them without compromising statistical power. Another limitation was the absence of serum cortisol evaluation at both time points, although sample collection itself may induce transient increases in glucocorticoids. Given the relatively small sample size and the number of cardiovascular variables assessed (ECG, blood pressure, echocardiographic indices, and stress scores), the study may have been underpowered to detect subtle physiological changes, and therefore minor effects of F3 cannot be ruled out. Wave fusion also represents a limitation, as overlapping E and A waves may have hindered precise diastolic function assessment by reducing the number of analyzable data points. Additionally, the absence of the animals’ owners during assessments may have influenced the results, highlighting the need to evaluate product effects in the presence of owners.

## Conclusion

Based on the results of this preliminary study, exposure to the synthetic feline facial pheromone analogue F3 in diffuser and spray formulations was associated with observable decreases in behavioral stress scores and did not produce observable short-term adverse cardiovascular effects in healthy cats. Long-term safety and effects in animals with pre-existing cardiac conditions were not evaluated. Further studies are warranted, particularly to explore potential sex-related differences and to confirm these findings under controlled conditions. These observations suggest that F3 may have potential for supporting stress management during veterinary cardiovascular assessments, but should be interpreted cautiously until validated in larger, randomized studies.

## Supplementary Information

Supplementary Material 1.

## Data Availability

The datasets used and/or analysed during the current study are available from the corresponding author on reasonable request. The supplementary material provides a description of the analyzed variables, including their type and the number of animals included in each analysis.

## Abbreviations

Ao: Aorta
BP: Blood pressure
CEUA: Ethics Committee on the Use of Animals
DAP: Diastolic arterial pressure
ECG: Electrocardiography
EDTA: Ethylenediaminetetraacetic acid
EF%: Ejection fraction
E/A: Mitral inflow E-to-A wave ratio
E′/A′: Tissue Doppler E′-to-A′ wave ratio
F3: Feline facial pheromone fraction 3
FeLV: Feline leukemia virus
FIV: Feline immunodeficiency virus
FS%: Fractional shortening
HR: Heart rate
IVRT: Isovolumetric relaxation time
IVSd: Interventricular septum thickness in diastole
IVSs: Interventricular septum thickness in systole
LA: Left atrium
LA/Ao: Left atrium-to-aorta ratio
LVFWd: Left ventricular free wall thickness in diastole
LVFWs: Left ventricular free wall thickness in systole
LVIDd: Left ventricular internal diameter in diastole
LVIDs: Left ventricular internal diameter in systole
MAP: Mean arterial pressure
MAPSE: Mitral annular plane systolic excursion
RR: Respiratory rate
SAP: Systolic arterial pressure
T0: Baseline evaluation without pheromone exposure
T1: Post-exposure evaluation to pheromone
UFMG: Federal University of Minas Gerais
