# Supplementary material for: Effects of a synthetic feline facial pheromone analogue (F3, Feliway®) on stress and cardiovascular parameters during clinical examination in cats: a prospective crossover study
Source: BMC Vet Res. 2026 May 26;22:470. doi: 10.1186/s12917-026-05511-x (PMC13471606; doi:10.1186/s12917-026-05511-x)
Supplement: Supplementary file 1 [file 12917_2026_5511_MOESM1_ESM.docx]

**Supplementary Table 1.** Variables included in the statistical analysis, type, and sample size (n)

| Variable | Type | n |
| --- | --- | --- |
| Name | Nominal qualitative | 21 |
| Breed | Nominal qualitative | 21 |
| Sex | Dichotomous qualitative | 21 |
| Age | Continuous quantitative | 21 |
| Weight | Continuous quantitative | 21 |
| Fiv | Dichotomous qualitative | 21 |
| Felv | Dichotomous qualitative | 21 |
| Dental tartar | Dichotomous qualitative | 21 |
| Periodontal alterations | Dichotomous qualitative | 21 |
| Mucous membranes | Ordinal qualitative | 21 |
| HR | Discrete quantitative | 21 |
| Murmur | Dichotomous qualitative | 21 |
| RR | Discrete quantitative | 21 |
| SAP Doppler | Discrete quantitative | 21 |
| SAP Oscillometric | Continuous quantitative | 21 |
| MAP Oscillometric | Continuous quantitative | 21 |
| DAP Oscillometric | Continuous quantitative | 21 |
| Femoral pulse | Ordinal qualitative | 21 |
| Body temperature | Continuous quantitative | 21 |
| Paw pads | Nominal qualitative | 21 |
| Muscle condition score | Ordinal qualitative | 21 |
| Body condition score | Ordinal qualitative | 21 |
| Thyroid aspect | Nominal qualitative | 21 |
| Stress (Observer 1) | Ordinal qualitative | 21 |
| Stress (Observer 2) | Ordinal qualitative | 21 |
| P-wave amplitude | Continuous quantitative | 21 |
| P-wave duration | Discrete quantitative | 21 |
| PR interval duration | Discrete quantitative | 21 |
| Q-wave amplitude | Continuous quantitative | 21 |
| R-wave amplitude | Continuous quantitative | 21 |
| R-wave duration | Discrete quantitative | 21 |
| S-wave amplitude | Continuous quantitative | 21 |
| ST segment deviation | Continuous quantitative | 21 |
| ST segment duration | Discrete quantitative | 21 |
| T-wave amplitude | Continuous quantitative | 21 |
| T-wave duration | Discrete quantitative | 21 |
| Min HR | Discrete quantitative | 21 |
| Mean HR | Discrete quantitative | 21 |
| Max HR | Discrete quantitative | 21 |
| IVSd | Continuous quantitative | 21 |
| IVSs | Continuous quantitative | 21 |
| LVPWd | Continuous quantitative | 21 |
| LVPWs | Continuous quantitative | 21 |
| LVEDV | Continuous quantitative | 21 |
| LVESV | Continuous quantitative | 21 |
| FS | Continuous quantitative | 21 |
| EF | Continuous quantitative | 21 |
| Ao | Continuous quantitative | 21 |
| LA | Continuous quantitative | 21 |
| LA/Ao | Continuous quantitative | 21 |
| Pulmonary flow | Continuous quantitative | 21 |
| GP pulmonary | Continuous quantitative | 21 |
| Aortic flow | Continuous quantitative | 21 |
| GP aortic | Continuous quantitative | 21 |
| Wave fusion | Dichotomous qualitative | 21 |
| E or EA wave | Continuous quantitative | 21 |
| A wave | Continuous quantitative | 11 |
| E/A ratio | Continuous quantitative | 11 |
| E-wave deceleration time | Continuous quantitative | 11 |
| E′ or EA′ wave | Continuous quantitative | 21 |
| A′ wave | Continuous quantitative | 11 |
| E′/A′ ratio | Continuous quantitative | 11 |
| IVRT | Continuous quantitative | 21 |
| MAPSE | Continuous quantitative | 21 |

*Consider: FIV: Feline immunodeficiency virus; FeLV: Feline leukemia virus; HR: Heart rate; RR: Respiratory rate; SBP: Systolic blood pressure; MAP: Mean arterial pressure; DBP: Diastolic blood pressure; MIN HR: Minimum heart rate; MEAN HR: Mean heart rate; MAX HR: Maximum heart rate; IVSd: Interventricular septum in diastole; IVSs: Interventricular septum in systole; LVPWd: Left ventricular posterior wall in diastole; LVPWs: Left ventricular posterior wall in systole; LVEDV: Left ventricular end-diastolic volume; LVESV: Left ventricular end-systolic volume; FS: Fractional shortening; EF: Ejection fraction; Ao: Aorta; LA: Left atrium; LA/Ao: Left atrium-to-aorta ratio; GP: Pressure gradient; E: Early diastolic transmitral flow velocity; A: Late diastolic transmitral flow velocity; E/A: Ratio between early and late diastolic transmitral flow; E′: Early diastolic mitral annular velocity; A′: Late diastolic mitral annular velocity; E′/A′: Ratio between early and late diastolic annular velocities; IVRT: Isovolumetric relaxation time; MAPSE: Mitral annular plane systolic excursion;
